# Design, Structural Characteristic and Antibacterial Performance of Silver-Containing Cotton Fiber Nanocomposite

**DOI:** 10.3390/bioengineering9120770

**Published:** 2022-12-05

**Authors:** Rasim Alosmanov, Irada Buniyat-zadeh, Mustafa Soylak, Azad Shukurov, Solmaz Aliyeva, Sinan Turp, Gulnara Guliyeva

**Affiliations:** 1 Department of Chemistry, Baku State University, Z. Khalilov Str. 23, AZ1148 Baku, Azerbaijan; 2 Technology Research & Application Center (ERU-TAUM), Erciyes University, Kayseri 38039, Turkey; 3 Department of Chemistry, Faculty of Sciences, Erciyes University, Kayseri 38039, Turkey; 4 Turkish Academy of Sciences (TUBA), Cankaya, Ankara 06670, Turkey; 5 “SOCAR Polymer” LLC, AZ5000 Sumqayit, Azerbaijan; 6 Scientific-Research Institute Geotechnological Problems of Oil, Gas and Chemistry, D. Aliyeva 227, AZ1010 Baku, Azerbaijan; 7 Department Chemical & Chemical Processing Technology, Tatvan Vocat High School, Bitlis Eren University, Bitlis 13000, Turkey; 8 Azerbaijan Republican Sanitary & Quarantine Center, AZ1009 Baku, Azerbaijan

**Keywords:** cotton fiber, chemical modification, silver-containing nanocomposite, antibacterial activity

## Abstract

In the present study, cotton fiber was treated with phosphorus trichloride in the presence of oxygen. As a result of the subsequent hydrolysis of modified cotton fibers, phosphorus-containing fragments with acidic groups and chlorine atoms were introduced onto their surface. Afterward, silver-containing composites based on raw and modified cotton fibers were prepared using the chemical reduction method. The obtained samples were characterized in detail by Fourier transform infrared spectroscopy, ultraviolet–visible spectroscopy, X-ray powder diffraction, as well as by thermogravimetric analysis, scanning electron microscopy, and energy-dispersive X-ray analysis. A comparative bioassay experiment of four samples for gram-negative (*Escherichia coli*) bacteria, gram-positive (*Staphylococcus aureus*) bacteria, and the fungus *Candida albicans* was carried out. These results showed the predominant antibacterial activity of the phosphorylated sample and the composite based on it. Thus, the development of these antibacterial cotton fibers using readily available reagents under relatively mild conditions could be used as potential industrial applications for the production of everyday medical textiles.

## 1. Introduction

The current generation, perhaps more often than all the previous ones, is faced with natural disasters, man-made disasters, epidemics, and other emergencies, which significantly increase the risk of adverse environmental effects on the human body. All this leads to the need to take all possible measures to prevent these undesirable phenomena and create more effective means of protecting the human body from pathogens [1,2]. From this point of view, the development of protective clothing materials with an antimicrobial finish is relevant. Such textiles, resulting in self-decontaminating fabrics, have potential benefits in terms of reduced disease transmission among hospital populations, protection against biological weapons, and other applications [3]. A wide range of chemicals and methods are currently available for the production of antibacterial textiles [4]. However, due to their toxicity, biocompatible and environmentally materials are preferred [5].

Cotton, as the most widely used natural fiber, attracts a lot of attention due to its breathability, softness, and degradability [5] but can act as a breeding ground for microorganisms. Moreover, when in contact with the human body, cotton fabrics can create an ideal environment for microbial growth due to their large surface area and hydrophilicity. The development of surface science and composite technology has made it possible to solve the above-mentioned disadvantages of cotton fabrics. In particular, more and more cotton fibers are being investigated for functionalization, whereby stable covalent bonds are formed between the fiber segments and chemical modification or grafting of the cotton surface is achieved, which leads to an improvement in its biological properties [6,7,8]. Hence, by incorporating various antimicrobial agents, such as silver nanoparticles (AgNPs) [9,10], copper and zinc oxides [11,12], (3-carboxypropyl) trimethylammonium chloride [13], and chitosan/titanium dioxide [14], different biofunctional cotton materials have been developed.

AgNPs are one of the most promising antimicrobial agents due to their more favorable properties, including a large specific surface area, high activity against a wide range of pathogens, low toxicity to human beings, low cost, and convenience for preparation and application [15]. Thus, AgNP-containing cotton materials are widely used for the manufacture of antimicrobial fabrics [16]. Typically, for the preparation of such materials, cotton is previously subjected to modification, as a result of which functional groups are chemically fixed onto its surface to provide the coordination of silver with cotton. The ability of some functional groups, such as hydroxyls, amines, thioether [9], carboxylate [17], and methyl [18], preliminarily attached to the cotton surface, to bind AgNPs has been reported. According to the results obtained, the nature of the functional groups determines the amount of AgNPs introduced into the cotton fiber, which characterized the antibacterial properties of the material.

This study aimed to obtain and characterize a new antibacterial Ag-containing nanocomposite based on functionalized cotton. For the first time, the functionalization of raw cotton fiber (cotton-OH) was carried out by using the reaction of oxidative chlorophosphorylation with the further hydrolysis of the obtained modifier. We have previously applied this method for the functionalization of butadiene rubber and graphite. It has been established that the final products comprise both phosphorus-containing groups and chlorine-containing fragments in their structure [19,20]. Thus, the modification reaction we have chosen makes it possible to introduce two types of functional groups into cotton-OH using a technique different from all previously known ones. The chemical structure of the functional groups contributes to the production of various AgNPs, which is the determining factor of the antibacterial properties of the obtained material.

In this work, the prepared samples were characterized using Fourier transform infrared (FTIR) spectroscopy, ultraviolet–visible (UV-Vis) spectroscopy, X-ray diffraction (XRD), thermogravimetric analysis (TGA), scanning electron microscopy (SEM), and energy-dispersive X-ray analysis (EDX). In addition, the comparative antibacterial activity of cotton-OH, functionalized cotton after modification, and Ag-containing composites was investigated against *Escherichia coli* (*E. coli*), *Staphylococcus aureus* (*S. aureus*), and *Candida albicans* (*C. albicans*).

## 2. Experimental Procedure

### 2.1. Materials

Cotton˗OH was purchased from the *Zardab* district (Azerbaijan). It was previously cleaned from mechanical impurities, washed with water and acetone, and dried under a vacuum. PCl_3_, CCl_4_, NaOH, AgNO_3_, and NaBH_4_ (all p.a.) were supplied by Gorex-Analyt GmbH and used without future purification. Oxygen was supplied to the reaction medium by purging through the concentrated H_2_SO_4_. All aqueous solutions for experiments were prepared using distilled water.

### 2.2. Modification of Cotton-OH

Modification of cotton-OH was carried out by oxidative chlorophosphorylation reaction and subsequent hydrolysis of the obtained product. Cotton-OH, previously cleaned and dried in a vacuum drying oven, was placed in a round-bottomed flask equipped with a thermometer, a reflux condenser, and a bubbler for oxygen supply. CCl_4_ (150 mL) was added to the flask containing cotton-OH (~2q) and kept for 24 h for preliminary swelling. Then, the first portion of the required amount of PCl_3_ was added to the reaction system and purged with oxygen at a rate of 7 L/h. The ratio of taken components was 1:5 (cotton-OH:PCl_3_). The reaction was carried out at room temperature for 3 h in a flow of oxygen, and when the next portion of PCl_3_ was added, the exothermic nature of the reaction appeared, as a result of which the temperature value increased to 33–35 °C. After its completion, the liquid phase was carefully separated from the resulting phosphochlorinated cotton fiber (cotton-(POCl_2_)(Cl)).

Then, ice–deionized water was added to cotton-(POCl_2_)(Cl) remaining in the flask for hydrolysis. This process was continued at room temperature for 4 h with gentle stirring. The resulting product was filtered and washed several times with distilled water until neutral pH was reached. Then, it was dried in air and placed under a vacuum at 30 °C. This sample was named cotton˗(PO(OH)_2_)(Cl).

### 2.3. Preparation of Silver-Containing Cotton Fiber Nanocomposite

The nanocomposite was prepared in situ by immobilizing AgNPs on cotton-OH and cotton˗(PO(OH)_2_)(Cl) using a simple method of chemical reduction of silver ions in the presence of sodium borohydride [21] (details in Supplementary material).

### 2.4. Characterization Techniques

FTIR measurements were performed on a Perkin Elmer 400 FTIR spectrometer (PerkinElmer Instrument, Washington, DC, USA) in normal transmission mode. UV-Vis spectrophotometer (SPECORD 210 PLUS, Analytik Jena, Jena, Germany) in a wavelength range of 200–800 nm was used to determine the absorption spectra. TGA was performed on a thermogravimetric analyzer (Perkin Elmer Diamond TG/DTA, PerkinElmer Instrument, Washington, DC, USA) at a heating rate of 10 °C/min from 30 °C to 800 °C in a nitrogen atmosphere. XRD analyses of the samples were performed using an X-ray diffractometer (Bruker AXS D8, Karlsruhe, Germany). The surface morphology of the cotton-OH, cotton˗(PO(OH)2)(Cl), and nanocomposite were observed using a field emission scanning electron microscope (Zeiss Gemini 500, Carl Zeiss SMT AG, Germany) after gold coating (about 5 nm thick). EDX spectroscopy was used to identify the elements present in the samples.

### 2.5. Antibacterial Test

The assessment of antibacterial and fungicidal properties was carried out according to the method “Determination of the sensitivity of microorganisms to antibacterial additives” [22] (details in the Supplementary material).

## 3. Results and Discussion

### 3.1. Description of the Synthesis Process

The modification of cotton˗OH is shown schematically in Supplementary Material Scheme S1a. Initially, as the main constituent part of cotton˗OH, cellulose reacts with PCl_3_ in the presence of O_2_ to form cotton-(POCl_2_)(Cl) with the phosphonyldichloride groups. Subsequently, the hydrolysis reaction of cotton-(POCl_2_)(Cl) was carried out, during which the phosphonyldichloride groups converted into phosphate groups, and the final product (cotton-(PO(OH)_2_)(Cl)) was obtained. Then, a large excess of NaBH_4_ reduced Ag^+^ ions to AgNPs, which were stabilized by the phosphate groups of cotton-(PO(OH)_2_)(Cl) (Supplementary Material Scheme S1b).

Silver-based composites based on cotton-OH were prepared by the same procedure (Supplementary Material Scheme S1c).

### 3.2. FTIR Spectroscopy

**Figure 1:**
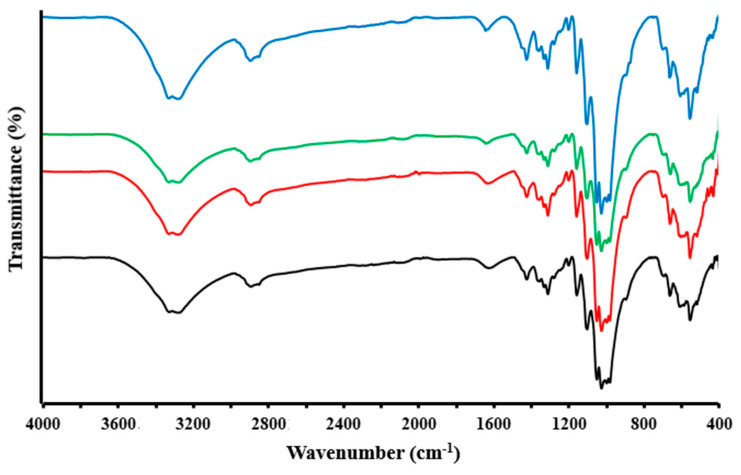
FTIR spectra of cotton−OH (black), cotton−OH−Ag (red), cotton−(PO(OH)_2_)(Cl) (green), and cotton−(PO(OH)_2_)(Cl) −Ag (blue).

The supposed chemical structure of the samples was confirmed by FTIR spectroscopy. FTIR spectra of cotton˗OH, cotton˗OH-Ag, cotton-(PO(OH)_2_)(Cl), and cotton-(PO(OH)_2_)(Cl)-Ag are shown in Figure 1 and Supplementary Material Figure S1.

Cotton fiber is usually characterized by several absorption bands. In the spectrum of the cotton-OH sample, a large absorption band at 3600–3000 cm^−1^ can be attributed to the free OH stretching vibration, as well as to intra- and intermolecular hydrogen bonds associated with the chemical structure of cellulose [23]. Near this region, in the range 3000–2800 cm^−1^, there are several narrow bands related to the stretching vibrations of the methylene and methine groups. Usually, in this region, a single absorption band can be observed for samples consisting only of cellulose [24,25,26]. The appearance of multiple absorption bands in this area in the original (initial) cotton is explained by the presence of pectin and waxes in its structure (pectin: 0.7–1.2%, waxes: 0.4–1.2%) [23]. The width of the band observed in the range of 1700–1500 cm^–1^ is because of not only the bending vibrations of H–OH bonds caused by the presence of adsorbed water [24] but also the peaks associated with asymmetric stretching vibrations of carboxyl groups in pectin acid and pectate [27,28]. The shoulder in the region of 1453–1440 cm^−1^ is associated with the presence of an absorption band of stretching of the O-CH_3_ bond (1445 cm^−1^) of pectin ester [27,28].

In the range of 600–1450 cm^−1^, specific and common bands appear, which are assigned to cellulose. The band at 1426 cm^−1^ is related to the CH_2_ bending of cellulose. The bands caused by the deformation of the OH groups of cellulose are located at 1334 and 1368 cm^−1^. The band at 1280 cm^−1^ refers to the stretching of the C=O and G ring, and the bands at 1160 and 1202 cm^−1^ refer to the symmetric and asymmetric stretching of the C-O-C bond. The bands from 1027 up to 1106 cm^−1^ indicate the vibrations of C-O, C-C, and C-H bonds in the ring and side groups of cellulose. The shoulder in the range of 1160–1135 cm^−1^ (peak at 1151 cm^−1^) is the result of the presence of pectin in the cotton structure [27], which is absent in “pure” cellulose [24]. A small peak at 888 cm^−1^ also applies to pectin [27]. The band observed at 897 cm^−1^ indicates the presence of β-glycosidic bonds between monosaccharides [23]. A broad absorption peak at 685–519 cm^−1^, against which a number of bands of different intensities appear, characterizes various vibrations of the pyranose ring and the bending vibrations of hydroxyl groups [29].

A comparative analysis of the spectra of the initial sample, and the silver-containing composite based on it, revealed some changes in the ranges of 1370–1360 cm^−1^ and 622–568 cm^−1^, which are explained by the fact that the hydroxyl groups located on the surface of cotton can bind silver nanoparticles [9]. The participation of hydroxyl groups in the stabilization of silver nanoparticles is confirmed by the conformational changes in the CH_2_ and CH groups associated with them, resulting in a shift of the peak from 2890 to 2888 cm^−1^. Along with this, changes are found in the range of 1560–1580 cm^−1^ (the peaks become more distinct). The shoulder at 1453–1440 cm^−1^ (a peak appears at 1448 cm^−1^) and peak at 888 cm^−1^ disappeared, which indicates the participation of pectin (in the cotton structure) in the binding of silver nanoparticles due to the presence of carboxyl groups in it [29]. The peak at 608 cm^−1^ in the spectrum of the composite confirms the presence of silver nanoparticles in its structure [30]. In addition, compared with the initial cotton, some differences are observed in the region of 500–600 cm^−1^, where, according to published investigations, metal–oxygen stretching vibrations are found [31].

The silver-containing sample has a shoulder at 545 cm^−1^ related to lattice vibrations of silver oxide [32], the formation of which is confirmed by a well-defined absorption band at 519 cm^−1^ [33] attributed to its (Ag_2_O) stretching vibrations.

The interpretation of the IR spectrum of cotton-(PO(OH)_2_)(Cl) became more complicated due to the low degree of conversion and the overlap of absorption bands. However, some differences were found between the IR spectra of the original sample and the modified product. According to the literature data, the absorption bands for P=O (1200–1400 cm^−1^), P=OH (820–1000 cm^−1^), and P-O-C (810–1190 cm^−1^) are characteristic for phosphorus-containing cellulose and cotton [34,35]. A detailed study of the cotton-(PO(OH)_2_)(Cl) spectra made it possible to establish differences (changes in the intensity of the peaks and their nature) at 1380–1347, 1180–1137, 980–1030, 920–875, 730–566, and 536–440 cm^−1^ regions, which confirms the modification of cotton. In addition, the peak at 492 cm^−1^ refers to the C-Cl bond, the formation of which is the result of an oxidative chlorophosphorylation reaction with the formation of chlorine radicals, which attack the macromolecular chain and subject it to chlorination [19]. In this case, in contrast to cotton-OH, the absorption band in the range of 1500–1700 cm^−1^ becomes not so wide and relatively clearly defined with two peaks at 1633 and 1645 cm^−1^. This can be explained by the fact that during oxidative chlorophosphorylation, the pectin component of cotton (the content of which in cotton is 0.7–1.2%) [23] is modified, turning into its phosphorus-containing derivative, and after washing the modified cotton, is removed from its composition. In other words, chemical modification leads to partial bioscouring.

In the spectrum of cotton-(PO(OH)_2_)(Cl)-Ag, changes are observed in the regions of 1387–1346 cm^−1^ (in contrast to cotton-(PO(OH)_2_)(Cl), two peaks are present) and 1180–1135 cm^−1^ (the absorption band becomes more intense and relatively narrow with a well-defined peak), which confirms the participation of phosphorus-containing groups in the binding of silver nanoparticles. In addition, in the region of 622–568 cm^−1^, changes are clearly defined, as in the case of a silver-containing composite based on the original cotton. This explains the binding of silver nanoparticles in the phosphorus-containing sample not only by its phosphorus-containing groups but also by the hydroxyl groups of the original cotton. The narrowing of the absorption bands in the region of 2856–2916 cm^–1^ indicates conformational changes in cellulose macromolecules as a result of the binding of silver nanoparticles by the modified sample.

The presence of silver nanoparticles in phosphorus-containing cotton is confirmed by new peaks at 1452, 1438, 1353, and 608 cm^−1^ [30,36], and the peak at 519 cm^−1^ also confirmed the presence of Ag_2_O in the sample. In contrast to the silver-containing composite based on the original cotton, the cotton-(PO(OH)_2_)(Cl)-Ag sample has a clear peak at 874 cm^−1^, which is attributed to AgCl [37]. This can be explained by the presence of C–Cl in the phosphorylated sample, which is also involved in the binding of silver nanoparticles.

### 3.3. UV–Visible Spectroscopy

**Figure 2:**
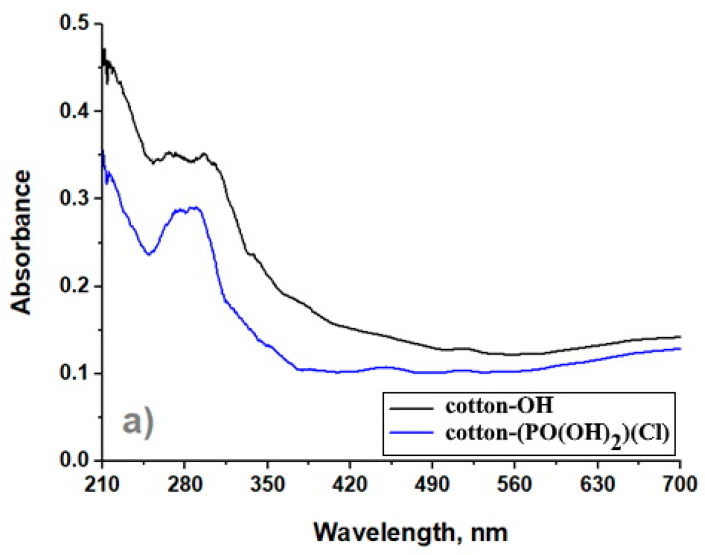

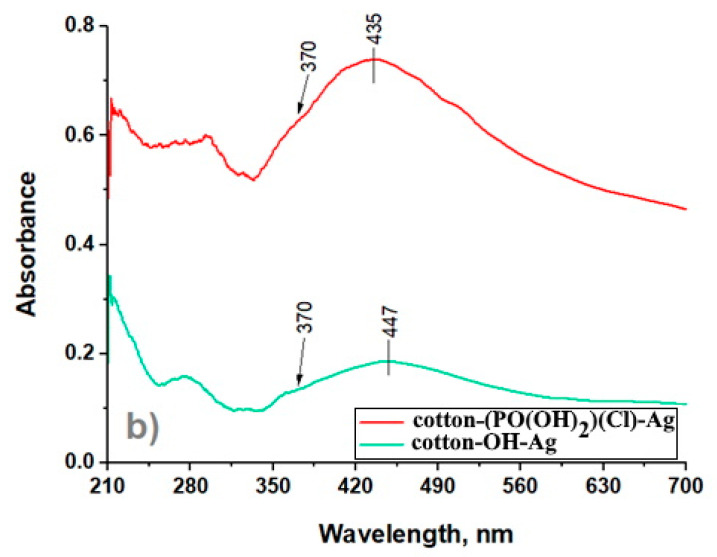
UV–visible spectra of samples: cotton-OH and cotton-(PO(OH)_2_)(Cl) (**a**) and cotton-OH-Ag and cotton-(PO(OH)_2_)(Cl)-Ag (**b**).

The UV–visible spectra of the cotton-OH and cotton-(PO(OH)_2_)(Cl) samples are shown in Figure 2a. As can be seen from the figure, the cotton-OH sample exhibits peaks in the UV region (between 200–300 nm). In the cotton-(PO(OH)_2_)(Cl) sample, the absorption of these peaks in the UV region is low. This indicates that the ability of cotton-OH to absorb UV radiation decreased after the oxidative chlorophosphorylation reaction [38].

Figure 2b shows the UV–visible spectrum of cotton-OH-Ag and cotton-(PO(OH)_2_)(Cl)-Ag samples. As can be seen from the figure, the surface plasmon resonance (SPR) peak of the silver nanoparticles can be observed in the spectrum of both samples. The peak intensity observed at 435 nm in the spectrum of the cotton-(PO(OH)_2_)(Cl)-Ag sample is greater than the peak intensity observed at 447 nm in the spectrum of cotton-OH-Ag. This result shows that more silver nanoparticles are formed on the cotton-(PO(OH)_2_)(Cl) surface, even though AgNO_3_ and NaBH_4_ used in the synthesis of cotton-OH-Ag and cotton-(PO(OH)_2_)(Cl)-Ag have the same concentration and the same nanoparticle synthesis time. In addition, according to Mie’s theory, the SPR peak corresponding to the AgNPs in both samples and the “shoulder” observed at 370 nm at that peak show that the AgNPs that formed were in the form of hollow spheres [39].

The Tauc equation was used to calculate the optical bandgap of cotton-OH-Ag and cotton-(PO(OH)_2_)(Cl)-Ag samples [40] (Supplementary Material Figure S2). As shown in Figure S2, the optical bandgap of cotton-(PO(OH)_2_)(Cl)-Ag (3.0 eV) is smaller than that of cotton-OH-Ag (3.8 eV). This result can be explained by the fact that in the cotton-(PO(OH)_2_)(Cl)-Ag sample, along with AgNPs, AgCl nanoparticles formed, which can lower the optical bandgap.

AgNPs, AgCl NPs, and Ag/AgCl nanocomposite were synthesized by Nehra and colleagues using the green method. It was determined that the bandgap of Ag/AgCl is 2.53 eV, the bandgap of AgNPs is 2.85 eV, and the bandgap of AgCl nanoparticles is 2.61 eV [37].

### 3.4. XRD Analysis

**Figure 3:**
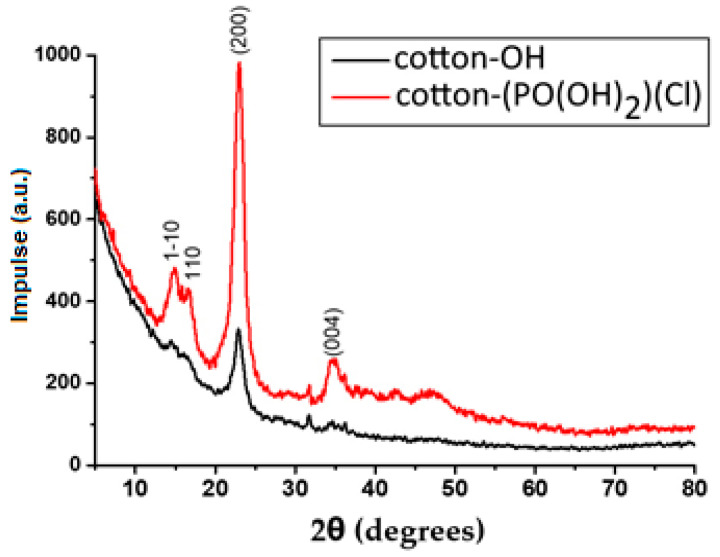
XRD spectra of cotton-OH and cotton-(PO(OH)_2_)(Cl).

XRD analysis was performed to determine how the oxidative chlorophosphorylation reaction and the formation of AgNPs alter the structure and packaging of cellulose macromolecules in cotton-OH. A comparison of the XRD spectra of cotton-OH and cotton-(PO(OH)_2_)(Cl) is shown in Figure 3.

As can be seen from the figure, the diffraction spectrum of cotton-OH has four main characteristic peaks corresponding to the cellulose allomorph Iβ at 14.57° (1–10), 16.64° (110), 22.89° (200), and 34.81° (004) 2θ [41]. Cotton-(PO(OH)_2_)(Cl) also shows a diffraction peak at nearly the same 2θ (14.65°, 16.96°, 23.00°, and 34.81°) values, indicating that oxidative chlorophosphorylation does not alter the crystalline form of the cellulose. However, the intensity of these peaks is greater than that of the cotton, indicating that the crystallization of cotton-(PO(OH)_2_)(Cl) is higher than that of cotton-OH. This can be explained by the fact that HCl, formed during the hydrolysis reaction of cotton-(POCl_2_)(Cl) for the synthesis of cotton-(PO(OH)_2_)(Cl), causes the process of acid hydrolysis [42], because of which amorphous domains are removed from cotton [43].

The XRD spectra of cotton-OH and cotton-OH-Ag are compared in Figure S3. As shown in the figure, cotton-OH and cotton-OH-Ag show diffraction (1–10), (110), (200), and (004) peaks at 14.65°, 16.96°, 23.00°, and 34.49°. This shows that in situ reduction does not change the crystalline shape of cotton [44].

Figure S4 compares the XRD spectra of cotton-(PO(OH)_2_)(Cl) and cotton-(PO(OH)_2_)(Cl)-Ag. As can be seen from the figure, the XRD pattern of cotton-(PO(OH)_2_)(Cl)-Ag also showed four main peaks at 14.78°, 16.57°, 22.76°, and 34.61° corresponding to the allomorph Iβ of cellulose. However, unlike cotton-(PO(OH)_2_)(Cl), in the diffractogram of cotton-(PO(OH)_2_)(Cl)-Ag, an additional peak corresponding to the cellulose II allomorph was observed at 2θ = 20.7° (110) as a “shoulder”, which indicates that structural changes occurred in the cellulose chain during the treatment of cotton-(PO(OH)_2_)(Cl) with NaBH_4_ (aqueous solution retains a certain amount of NaOH) and AgNO_3_ [45]. The peaks observed in the XRD spectrum of cotton-(PO(OH)_2_)(Cl)-Ag at values 29.13°, 30.88°, 45.21°, and 68.00° of 2θ may correspond to AgCl nanoparticles [46].

It should be noted that the peaks of AgNPs (Ag_2_O for cotton-OH-Ag and cotton-(PO(OH)_2_)(Cl)-Ag) are not visible in the XRD spectra of both cotton-OH-Ag and cotton-(PO(OH)_2_)(Cl)-Ag due to their low intensity. This can be explained by the high dispersion of AgNPs in organic substrates and the low sensitivity of XRD to the detection of metals in organic substrates [47]. A similar situation was observed in palladium nanoparticles and a covalent organic polymer-4-based material [48] and surface dielectric barrier discharge plasma-synthesized cotton and AgNP-based material [47].

Crystallite size and Segal crystallinity index (CI)

The crystal size of cellulose-based materials is usually calculated by researchers from the Scherrer formula based on the parameters of the dominant (200) peak [49].

(1) $$D=\frac{k\lambda }{\beta cos\theta }\;$$

where *D* is the crystallite size, *K* is the Braggs constant (0.9), *β* is the full width at half maximum (FWHM), *θ* is the Bragg angle, and *λ* is the X-ray wavelength (1.54056 Å).

Segal CI (%) is calculated by the following equation [50]:

(2) $$CI=\frac{I_{t}-I_{am}}{I_{t}}\times 100\%\;\;$$

where *I_t_* is the total height of the peak (200) for cellulose *Iβ, I_am_* is the minimum intensity between the main (200) peaks and the secondary (1–10) and (110) peaks.

The parameters of the diffraction peaks for the calculation of the crystallite size and Segal CI were determined by the “OriginPro 2015” software. The results obtained are given in Table S1. As can be seen from the table, the value of the Segal CI parameters of the samples changes in the direction: cotton-OH> cotton˗OH- Ag> cotton-(PO(OH)_2_)(Cl)> cotton-(PO(OH)_2_)(Cl)-Ag. An increase in the Segal CI parameter is usually observed upon hydrolysis of amorphous cellulose. This is because the broken cellulose chains have more freedom to create more organized structures [51].

### 3.5. Thermal Analysis

The TG/DTG/DTA curves taken for cotton-OH, cotton˗OH-Ag, cotton-(PO(OH)_2_)(Cl), and cotton-(PO(OH)_2_)(Cl)-Ag samples in a nitrogen medium in dynamic mode are shown in Figure S5. The parameters determined as a result of the analysis of these data are given in Table 1.

An analysis of the TG curves shows that the non-isothermal destruction of the studied samples proceeds through several successive decomposition processes accompanied by weight loss. At the first stage, up to 135 °C, a weight loss in samples by about 3–7% was observed, depending on the type of sample. An analysis of the DTA curves shows that at this stage, the mass loss is endothermic and can be explained by the removal of physically adsorbed water.

The next stage of decomposition is recorded in the temperature range of 250–420 °C and is the main stage of sample decomposition. An analysis of the DTA curves shows that in this case the process is accompanied by an exothermic effect. Further, the degradation process proceeds at a low rate and a slight mass loss. For all of the samples, the presence of residual mass at 600 °C is noted in Table 1.

As can be seen from Table 1, the values of T_onset_ and T_max_ (the temperature corresponding to the maximum degradation rate, according to DTG curves °C) for cotton-OH are 313 and 351 °C, respectively. For the cotton-(PO(OH)_2_)(Cl) sample, the corresponding parameters decreased, which indicates a decrease in thermal stability. The FTIR data show that the chemical modification results in the partial removal of non-cellulosic components, such as pectin and wax, from the original cotton (cotton-OH). This is also confirmed by XRD analysis, which shows that chemical modification leads to the removal of amorphous domains from cotton. According to published data [23], reducing the content of non-cellulosic components in cotton improves thermal stability and not vice versa. Perhaps the reason is related to oxidative chlorophosphorylation, which introduces the corresponding functional groups into the cellulose matrix (see the scheme), the presence of which leads to a partial decrease in the thermal stability of the resulting sample. In addition, the results of XRD analysis (Table S1) show that the reaction of oxidative chlorophosphorylation leads to the breaking of the macromolecular chains of cellulose. This may also be a reason for the decrease in the thermal stability of the sample.

In silver-containing samples, in comparison with the initial ones, an increase in thermal stability can be observed. The increase in thermal stability by the introduction of AgNPs into the original cotton was confirmed in [9]. The observed improvement in the thermal stability of the cotton-(PO(OH)_2_)(Cl)-Ag sample compared to cotton-(PO(OH)_2_)(Cl) can be explained as follows. First, silver nanoparticles in the modified sample (cotton-(PO(OH)_2_)(Cl)-Ag) form compounds such as AgCl (according to FTIR and XRD analysis), which are not intrinsic for the cotton-(PO(OH)_2_)(Cl) sample. The cotton-(PO(OH)_2_)(Cl) sample contains bonds such as C–Cl, which form relatively easily volatile compounds. Second, the presence of silver nanoparticles effectively restricts the motion of molecular segments in cellulose, which is accompanied by an increase in thermal stability of cotton-(PO(OH)_2_)(Cl)-Ag.

### 3.6. SEM and EDX Micrographs

**Figure 4:**
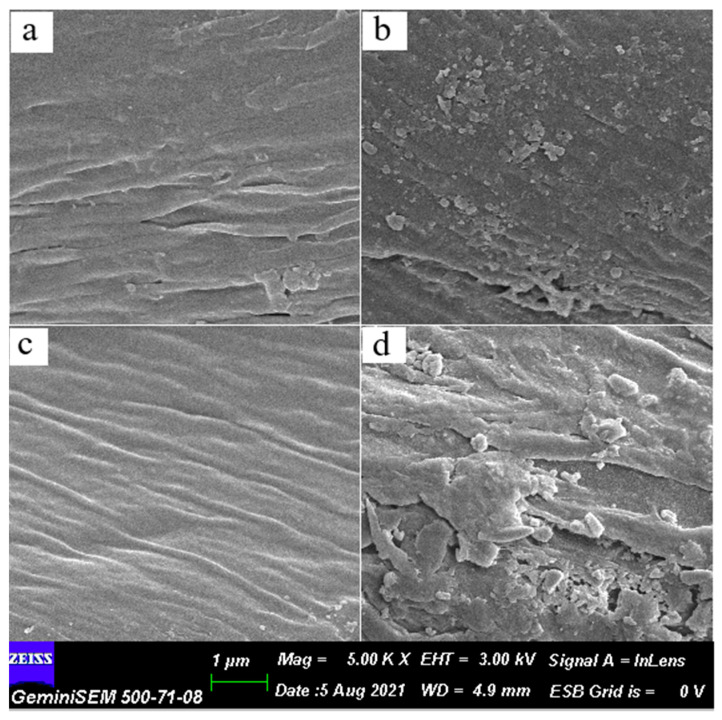

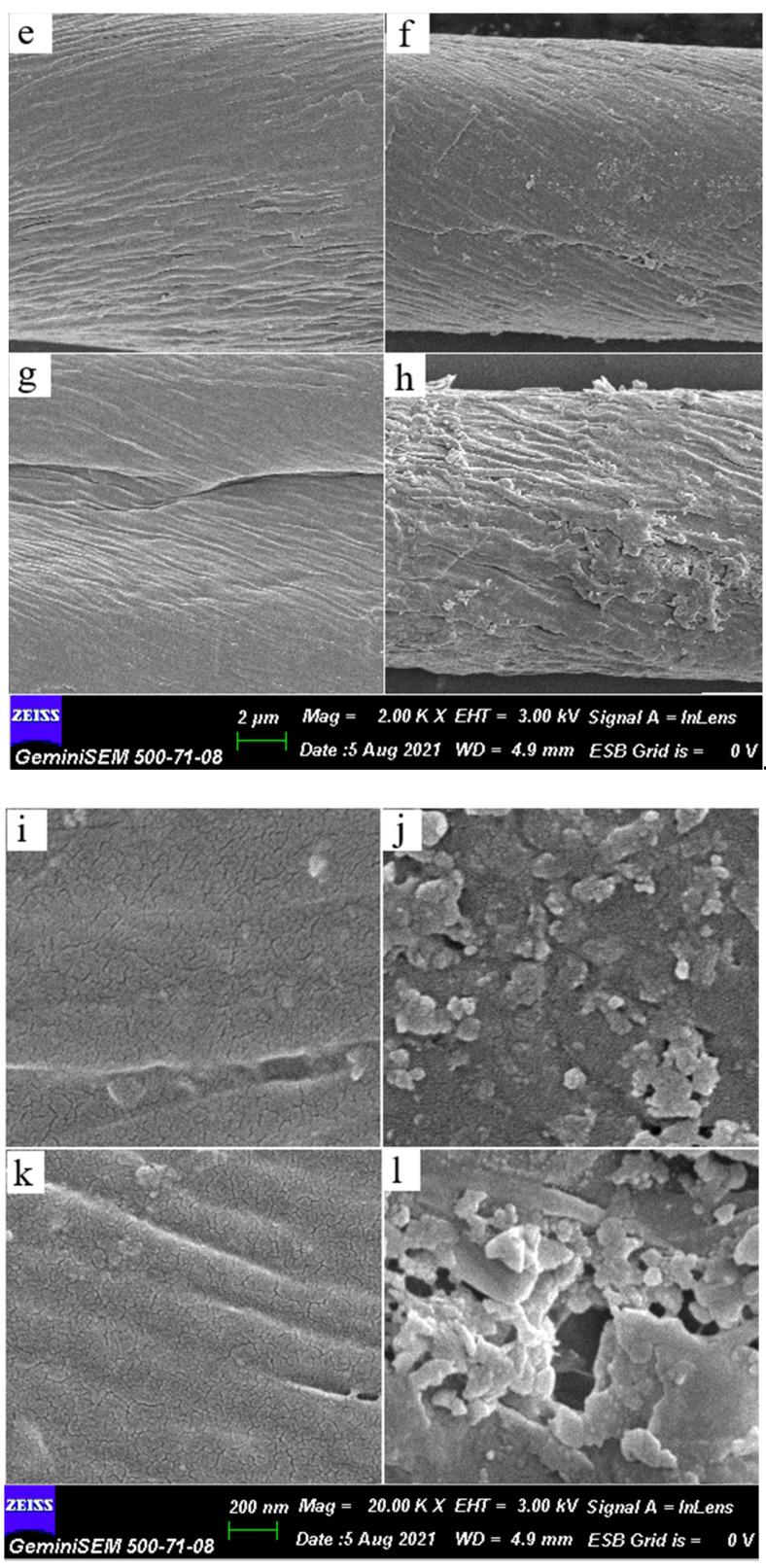
SEM images of cotton-OH (**a**,**e**,**i**); cotton˗OH-Ag (**b**,**f**,**j**); cotton-(PO(OH)2)(Cl) (**c**,**g**,**k**); and cotton-(PO(OH)2)(Cl)-Ag (**d,h,l**). Scale bars are 1 μm in (**a**–**d**), 2 μm in (**e**–**h**), and 200 nm in (**i**–**l**).

Figure 4 presents SEM images of the studied samples. As can be seen from the figure, the cotton-OH sample (Figure 4a,e,i) has a rough surface, which can be explained by the presence of non-cellulose components such as pectin and wax. Compared to cotton-OH, the surface of the chemically modified sample (cotton-(PO(OH)_2_)(Cl)) on the SEM image (Figure 4c,g,k) becomes less rough and smoother, which indicates the removal of pectin and wax from the structure of the sample during the modification process. Morphological changes in silver-containing samples (Figure 4b,f,j for cotton-OH-Ag and Figure 4d,h,l for cotton-(PO(OH)_2_)(Cl)-Ag), compared with the original ones, are pronounced, i.e., the presence of AgNPs on the surface of the samples is clearly visible, especially for the sample obtained that was based on chemically modified cotton.

EDX spectra were recorded to confirm the presence of Ag in the samples (Figure S6). A characteristic signal for Ag was detected at 3 kV and the very low intensity of the Ag peak may be associated with a low concentration of loaded Ag-containing nanoparticles.

The quantitative content of the elements is given in Table S2. As can be seen from these data, compared with the initial sample (cotton-OH), the oxygen concentration in the modified sample (cotton-(PO(OH)_2_)(Cl)) increases by approximately ~2%, which indicates the introduction of functional groups into the cellulose matrix. In addition, the concentration of silver in both silver-containing samples was in the amount of 0.38 (cotton-OH-Ag) and 0.42% (cotton-(PO(OH)_2_)(Cl)-Ag), respectively. Approximately, the same amount of Ag has been identified in amine-containing cotton [9].

### 3.7. Antibacterial Properties

Table 2 presents the results of the samples’ antibacterial activity. For comparison, pure cotton was also subjected to bio-analysis, which showed the absence of antibacterial activity, which is consistent with the available literature information [9]. Conversely, the sample of cotton-OH-Ag has definite antibacterial properties, which are due to the presence of silver nanoparticles in the composite [30]. The sample of cotton-(PO(OH)_2_)(Cl) also has special antibacterial properties, which can be explained by the presence of functional groups in the sample, especially chlorine atoms [52]. The composite that was prepared based on the phosphorus-containing sample (cotton-(PO(OH)_2_)(Cl)-Ag) has the best activity against all studied bacteria. The reason for this behavior cannot be explained by the presence of only functional groups or only by the presence of silver particles in the sample. This is because a sample containing only functional groups does not have such high activity. On the other hand, the silver concentration in the sample prepared from phosphorylated cotton is not much different from its concentration in the sample obtained from pure cotton. The antibacterial properties of the sample cotton-(PO(OH)_2_)(Cl)-Ag show the best effect can be explained by two reasons. Firstly, this is due to the activity of other silver compounds in modified cotton, especially AgCl, and, as it is known from the literature, AgCl particles have sufficient antibacterial properties [36]. Second, this effect can be caused by a more uniform distribution of silver particles in the sample due to the presence of functional groups (according to the SEM results). Thus, the maximum antibacterial properties of the sample are the result of synergistic effects.

## 4. Conclusions

Phosphonyldichloride groups and chlorine atoms were introduced into cotton-OH by the reaction of oxidative chlorophosphorylation. The subsequent hydrolysis of the modified product (cotton-(POCl_2_)(Cl)) converted the phosphonyldichloride groups into the corresponding acid groups. Silver-containing composites based on cotton-OH and cotton-(PO(OH)_2_)(Cl) were obtained by chemical reduction. Spectroscopic analysis, including FT-IR and UV-Vis techniques, along with X-ray diffraction analysis, TGA, and SEM-EDS, were used to characterize the structure of the samples in detail.

The following results were revealed:

- As a result of the chemical modification of the cotton-OH sample, a low degree of conversion could be observed, and the reaction product contained phosphate groups and chlorine atoms. In addition, non-cellulose compounds (pectin and wax) were removed from cotton-OH as amorphous domains, resulting in the higher crystallization of cotton-(PO(OH)_2_)(Cl) than cotton-OH. It should also be noted that after modification, the surface of the sample (cotton-(PO(OH)_2_)(Cl)) became less rough and smoother and its thermal stability decreased.
- Silver-containing particles in the cotton-OH-Ag composite were present in the form of zero-valent metal and oxide, and, in the cotton-(PO(OH)_2_)(Cl)-Ag composite, in addition to the above, AgCl particles were present. In general, the amount of silver nanoparticles in the cotton-(PO(OH)_2_)(Cl)-Ag composite was slightly higher than in cotton-OH-Ag, and they were well dispersed. In both composites, silver nanoparticles had the shape of hollow spheres. In silver-containing samples, in comparison with the original sample, an increase in thermal stability could be observed.

The antibacterial activity of the samples was tested against gram-negative (*E. coli*) bacteria, gram-positive (*S. aureus*) bacteria, and the fungus *C. albicans*. It was confirmed that the composite prepared based on a modified cotton sample (cotton-(PO(OH)_2_)(Cl)-Ag) had the highest activity against all tested bacteria. This effect was the result of the presence of various silver-containing nanoparticles (Ag, Ag_2_O and AgCl) and their uniform distribution.

## Figures and Tables

**Table 1:** The thermal parameters obtained from the TG/DTG curves of the investigated samples.

Samples	Stage II		Residual Mass at 600 °C, %
	DTG_max_	T_onset_	
Cotton-OH	351.0	313.0	18.00
Cotton-(PO(OH)_2_)(Cl)	314.8	285.0	24.74
Cotton˗OH-Ag	365.8	341.0	10.18
Cotton-(PO(OH)_2_)(Cl)-Ag	357.8	319.0	19.76

**Table 2:** The inhibition of bacterial growth by samples.

Samples	Cotton-OH (Blank)	Cotton-OH-Ag	Cotton-(PO(OH)_2_)(Cl)	Cotton-(PO(OH)_2_)(Cl)-Ag
*Escherichia coli*	0	27	8	150
*Staphylococcus aureus*	0	48	10	150
*Candida albicans*	0	24	26	150

## Data Availability

All data generated or analyzed during this study are included in this published article (and its Supplementary Information Files).

## Supplementary Materials

The following supporting information can be downloaded at: https://www.mdpi.com/article/10.3390/bioengineering9120770/s1, Scheme S1: Modification of cotton˗OH (a) and preparation of composites: cotton-OH-Ag (b), cotton-(PO(OH)_2_)(Cl)-Ag (c); Figure S1 FTIR spectra of cotton˗OH (black), cotton˗OH-Ag (red), cotton-(PO(OH)_2_)(Cl) (green) and cotton-(PO(OH)_2_)(Cl)-Ag (blue) in the different range; Figure S2 The optical bandgap for cotton˗OH-Ag and cotton-(PO(OH)_2_)(Cl)-Ag; Figure S3 XRD spectra of cotton-OH and cotton-OH-Ag; Figure S4 XRD spectra of cotton-(PO(OH)_2_)(Cl) and cotton-(PO(OH)_2_)(Cl)-Ag; Figure S5. TG/DTG and DTA curves of samples cotton-OH (black line), cotton-(PO(OH)_2_)(Cl) (green line), cotton˗OH-Ag (red line) and cotton-(PO(OH)_2_)(Cl)-Ag (blue line); Figure S6 EDX image for samples coated by Ag-containing nanoparticle: cotton˗OH-Ag (a) and cotton-(PO(OH)_2_)(Cl)-Ag (b); Table S1. Crystalline size and Segal CI of cotton-OH, cotton˗OH-Ag, cotton-(PO(OH)_2_)(Cl) and cotton-(PO(OH)_2_)(Cl)-Ag; Table S2. EDX Data of Different Cotton Fibers.
