# Supplementary material for: Design, Structural Characteristic and Antibacterial Performance of Silver-Containing Cotton Fiber Nanocomposite
Source: Bioengineering (Basel). 2022 Dec 5;9(12):770. doi: 10.3390/bioengineering9120770 (PMC9774151; doi:10.3390/bioengineering9120770)
Supplement: Supplementary file 1 [file bioengineering-09-00770-s001.zip › bioengineering-2048407-supplementary.pdf]

Supplementary material

DESIGN, STRUCTURAL CHARACTERISTIC AND ANTIBACTERIAL PERFORMANCE OF SILVER-CONTAINING COTTON FIBER NANOCOMPOSITE

Rasim Alosmanov[1]; Irada Buniyat-zadeh[1]; Mustafa Soylak[2][3][4]; Azad Shukurov[5]; Solmaz Aliyeva[6]; Sinan Turp[7]; Gulnara Guliyeva[8]

Rasim Alosmanov (corresponding author)

Department of Chemistry, Baku State University, Z. Khalilov Str. 23, AZ1148 Baku, Azerbaijan

+994-50-3780183; r_alosmanov@rambler.ru

[1] Department of Chemistry, Baku State University, Z. Khalilov Str. 23, AZ1148 Baku, Azerbaijan

[2] Technology Research & Application Center (ERU-TAUM), Erciyes University, 38039 Kayseri-Turkey

[3] Erciyes University, Faculty of Sciences, Department of Chemistry, 38039 Kayseri, Turkey

[4] Turkish Academy of Sciences (TUBA), Cankaya, Ankara, Turkey

[5] “SOCAR Polymer” LLC, AZ5000, Sumqayit, Azerbaijan

[6] Scientific-Research Institute Geotechnological Problems of Oil, Gas and Chemistry, D.Aliyeva 227, AZ1010, Baku, Azerbaijan

[7] Bitlis Eren Univ, Tatvan Vocat High Sch, Dept Chem & Chem Proc Technol, Bitlis, Turkey

[8] Azerbaijan Republican Sanitary & Quarantine Center, AZ1009, Baku, Azerbaijan

1. Preparation of silver-containing cotton fiber nanocomposite (cotton-OH-Ag, cotton-(PO(OH)$_2$)(Cl)-Ag)

Samples (cotton-OH or cotton-(PO(OH)$_2$)(Cl) (0.1 q) were placed in a flask containing 50 ml of $NaBH_4$ solution (2.0 mM). This was immersed in an ice-bath and under intensive stirring 16.6 ml of $AgNO_3$ solution (1.0 mM) was added drop by drop. When the entire amount of the silver salt was added to the solution, it acquired a characteristic bright yellow color. The obtained cotton-OH-Ag and cotton-

$(PO(OH)_2)(Cl)$-Ag were thoroughly rinsed with deionized water, then dried in the air, and kept in a desiccator for further characterization.

2. Antibacterial test

We used strains of gram-negative bacteria, *E. coli* ATCC®25922; gram-positive bacteria, *S. aureus* ATCC®25923; and fungus *C. albicans* ATCC®90028; and culture media from «Liofilchem®» (Italy) agar Mueller Hinton, Saburo, Endo, B.Parker, MacConkey.

The bacteria were cultured in Liofichem® trypto-soy broth under aerobic conditions at 37°C for 24 hours. Equal volumes of bacterial suspension (1 ml each) were layered on Petri dishes with selective medium for each pathogen using a standard inoculum corresponding to 0.5 density by McFarland standard and containing approximately 1.5×10^8 colony forming units (CFU)/ml. The partially closed dishes were placed into a drying box at room temperature for 15 minutes, after which the test samples were put into them. The samples were rolled into balls, each ~ 5 mm in diameter and ~ 0.01 g in weight. The dishes were incubated at 37°C for 24 hours. The degree of sensitivity of microorganisms was determined by measuring the diameter zone of inhibition (in mm).

3. Scheme of the modification of cotton-OH and preparation of composites

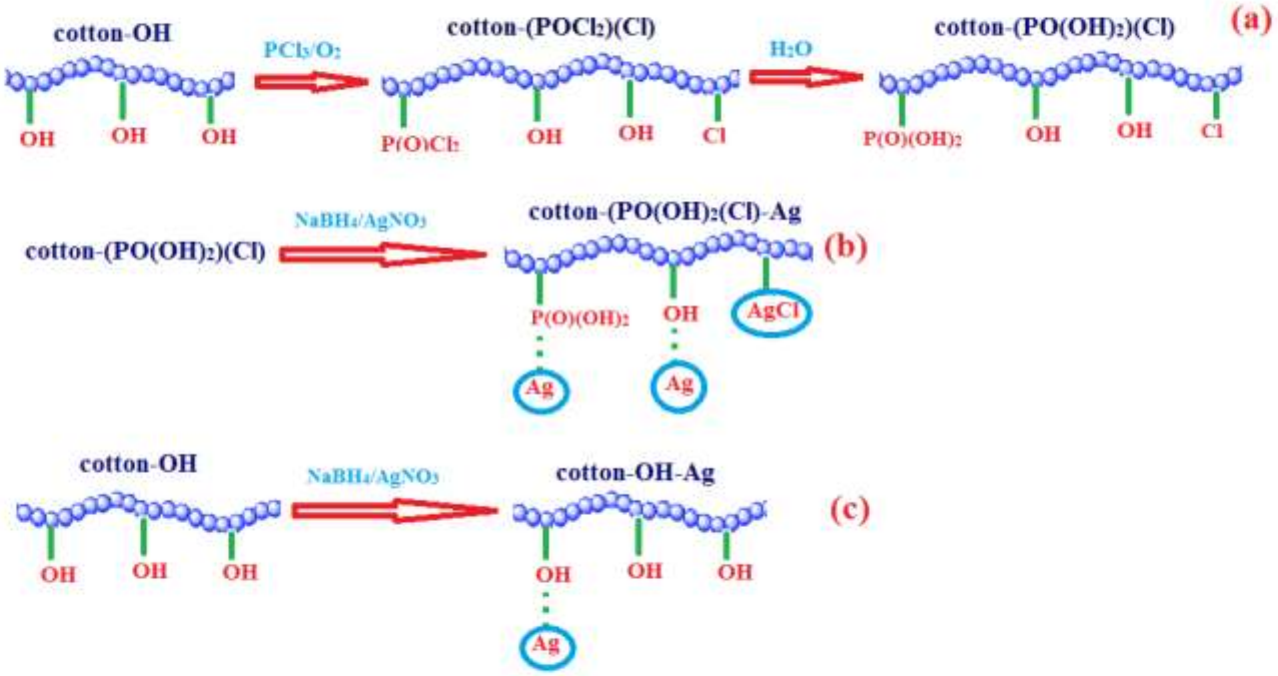

Scheme S1. Modification of cotton-OH (a) and preparation of composites: cotton-OH-Ag (b), cotton-$(PO(OH)_2)(Cl)$-Ag (c)

4. FTIR spectra of the samples in the different range

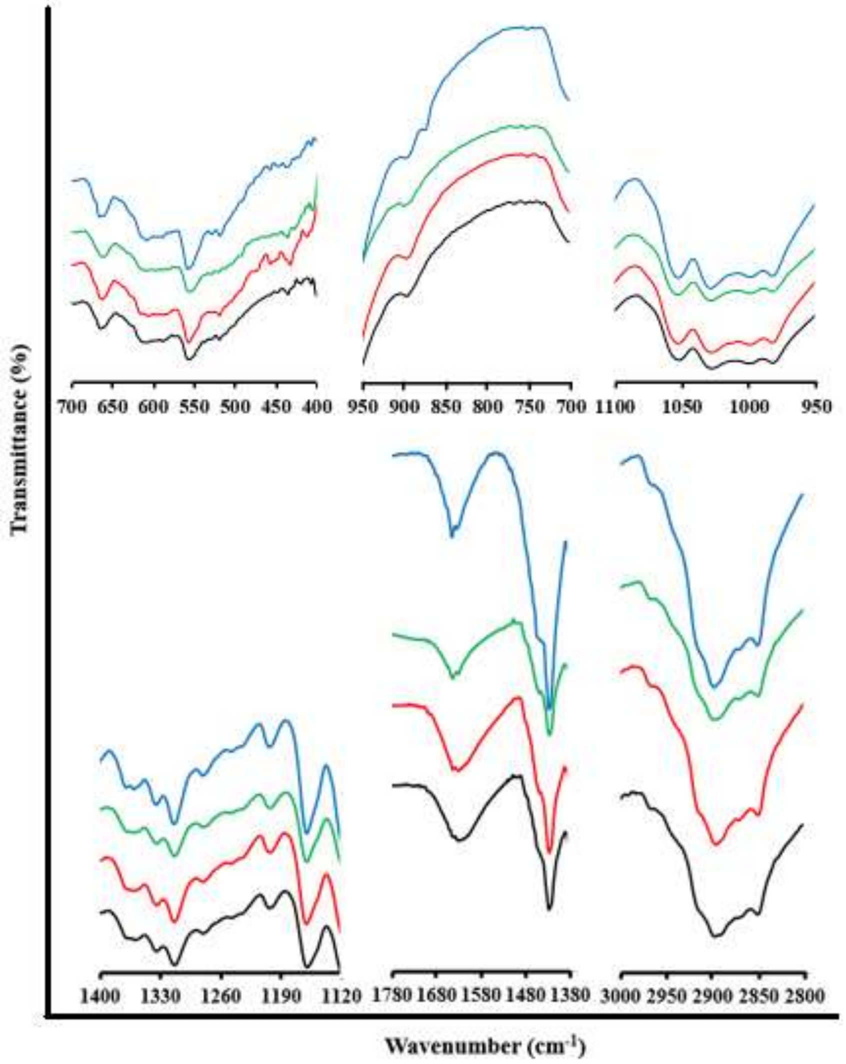

Figure S1. FTIR spectra of cotton-OH (black), cotton-OH-Ag (red), cotton-$(PO(OH)_2)$(Cl) (green) and cotton-$(PO(OH)_2)$(Cl)-Ag (blue) in the different range

5. The optical bandgap for cotton-OH-Ag and cotton-$(PO(OH)_2)$(Cl)-Ag

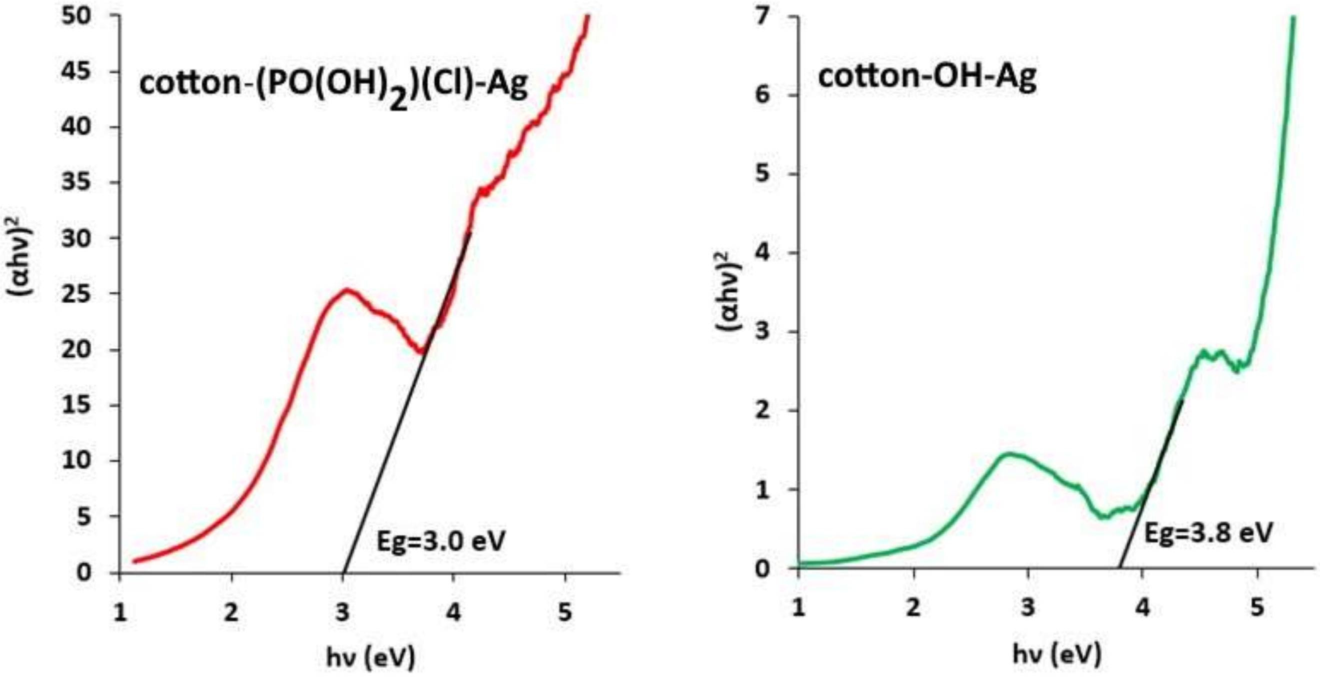

Figure S2. The optical bandgap for cotton-OH-Ag and cotton-$(PO(OH)_2)$(Cl)-Ag

6. XRD spectra of cotton-OH and cotton-OH-Ag

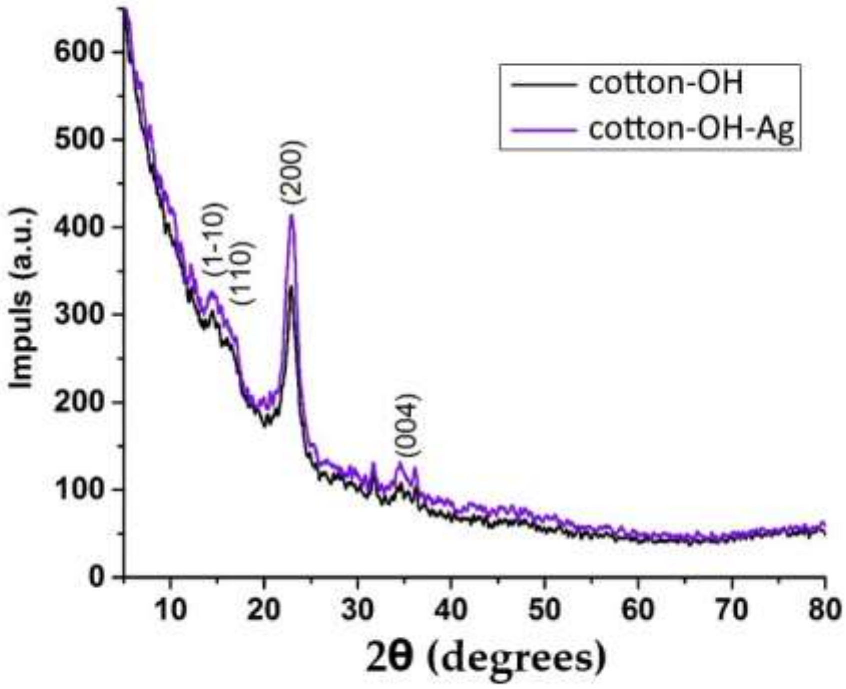

Figure S3. XRD spectra of cotton-OH and cotton-OH-Ag

7. XRD spectra of cotton-$(PO(OH)_2)$(Cl) and cotton-$(PO(OH)_2)$(Cl)-Ag.

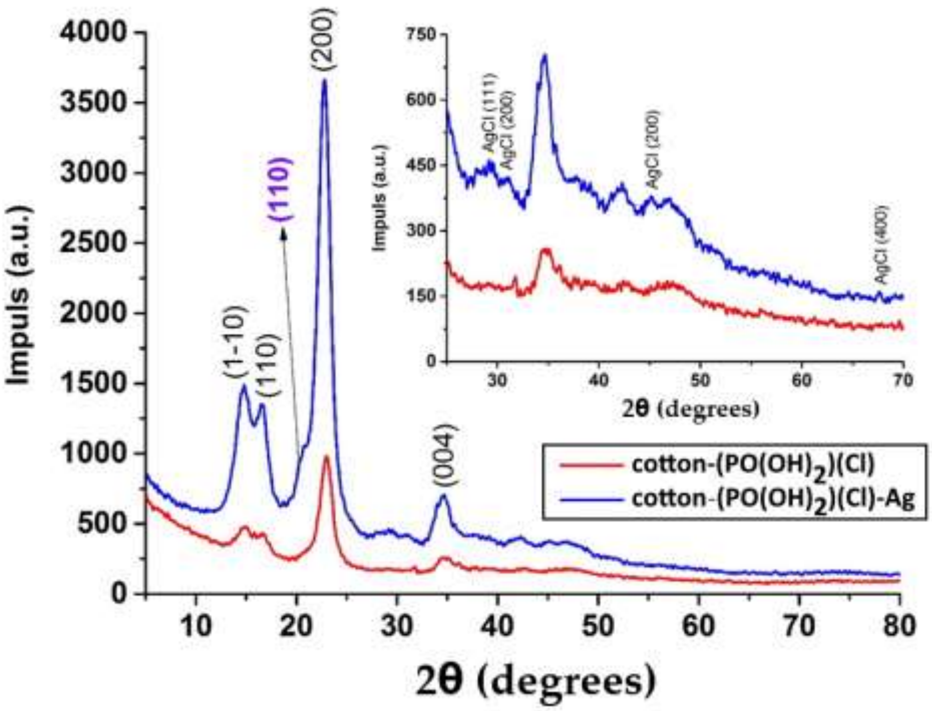

Figure S4. XRD spectra of cotton-$(PO(OH)_2)(Cl)$ and cotton-$(PO(OH)_2)(Cl)$-Ag

8. Crystalline size and Segal CI of the samples

Table S1. Crystalline size and Segal CI of cotton-OH, cotton-OH-Ag, cotton-$(PO(OH)_2)(Cl)$ and cotton-$(PO(OH)_2)(Cl)$-Ag.

Sample	2θ	Intensity	I_t	FWHM	I_{am}	D (nm)	Segal CI (%)
cotton-OH	14.57<br>16.64<br>22.89	301.70<br>260.60<br>332.64	31.60<br>23.33<br>176.43	0.72<br>0.46<br>1.19	196.10	7.12	41.05
cotton-OH-Ag	14.65<br>16.96<br>23.00	323.56<br>273.05<br>413.14	49.44<br>40.76<br>247.59	0.82<br>0.68<br>1.39	207.13	6.09	49.86
cotton-$(PO(OH)_2)(Cl)$	14.96<br>16.70<br>23.03	480.94<br>426.11<br>979.90	156.33<br>128.75<br>751.39	1.42<br>1.34<br>1.37	257.10	6.18	73.76

cotton-(PO(OH)$_2$)(Cl)-Ag	14.78<br>16.57<br>22.76	1476.82<br>1354.52<br>3663.80	915.74<br>813.50<br>3192.40	2.13<br>1.48<br>1.47	642.90	5.76	82.45

9. TG/DTG and DTA curves of samples

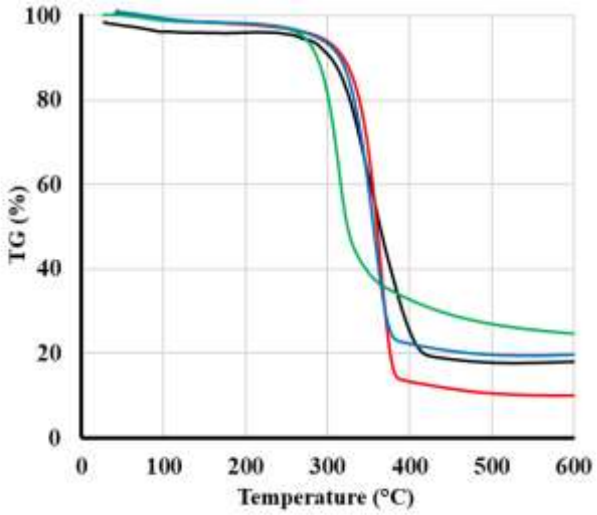

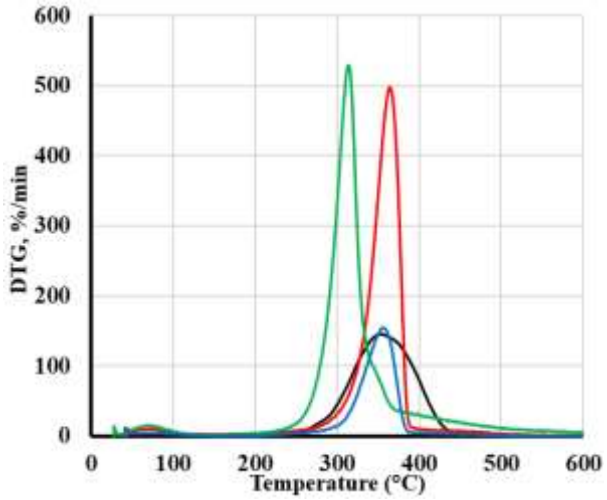

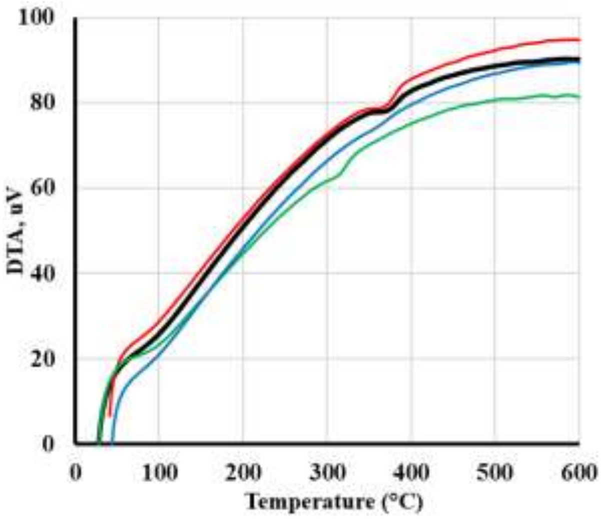

Figure S5. TG/DTG and DTA curves of samples cotton-OH (black line), cotton-(PO(OH)$_2$)(Cl) (green line), cotton-OH-Ag (red line) and cotton-(PO(OH)$_2$)(Cl)-Ag (blue line)

10. SEM images of the samples

11. EDX image for samples coated by Ag-containing nanocomposites

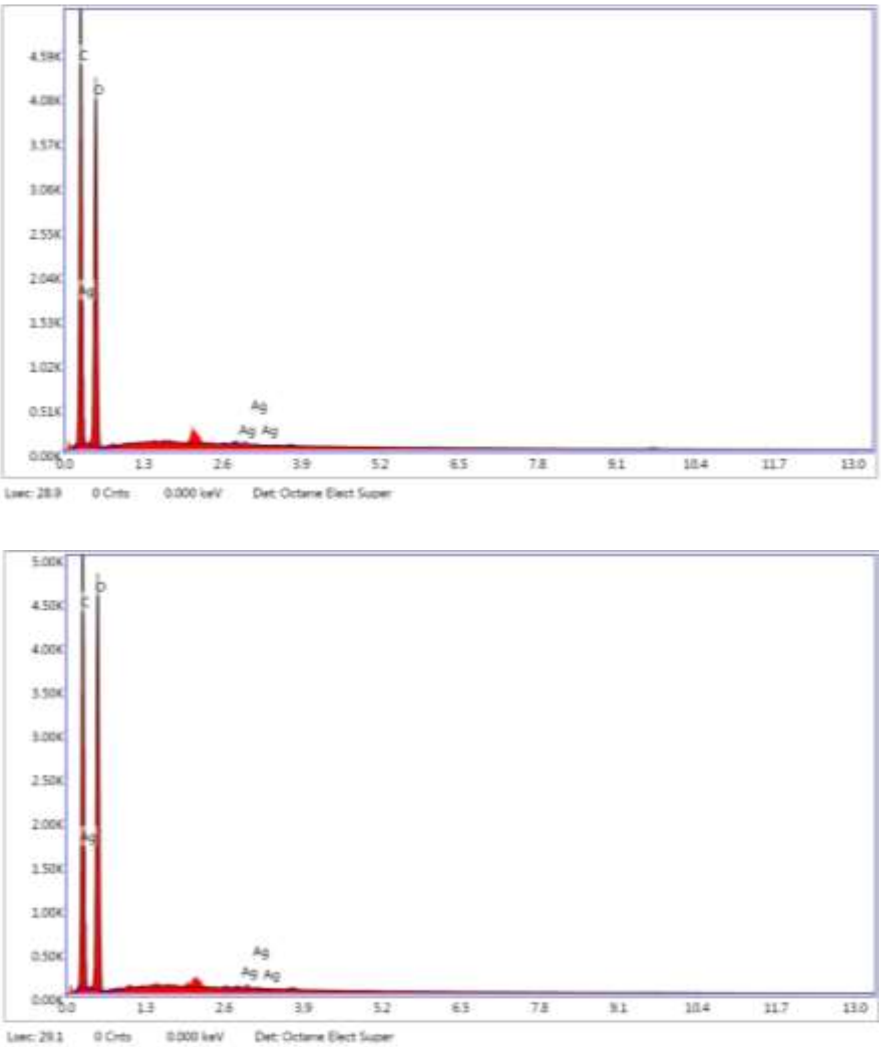

Figure S6. EDX image for samples coated by Ag-containing nanoparticle: cotton-OH-Ag (a) and cotton-($PO(OH)_2$)(Cl)-Ag (b).

12. The quantitative content of elements

Table S2. EDX Data of Different Cotton Fibers

Samples	C, %	O, %	Ag, %
cotton-OH	50.89	49.11	-
cotton-OH-Ag	47.32	52.31	0.38
cotton-($PO(OH)_2$)(Cl)	49.04	50.96	-
cotton-($PO(OH)_2$)(Cl)-Ag	46.28	53.30	0.42
